# Association of the hemoglobin glycation index with cardiovascular and all-cause mortality in individuals with hypertension: findings from NHANES 1999–2018

**DOI:** 10.3389/fendo.2024.1401317

**Published:** 2024-06-10

**Authors:** Qing Shangguan, Jingqi Yang, Bin Li, Huaigang Chen, Liu Yang

**Affiliations:** ^1^ Department of Cardiology, Jiangxi Provincial People’s Hospital, The First Affiliated Hospital of Nanchang Medical College, Nanchang, China; ^2^ Medical College of Nanchang University, Nanchang, China

**Keywords:** HGI, CVD, all-cause, mortality, hypertension, gender differences

## Abstract

**Background:**

This study examines the association between Hemoglobin Glycation Index (HGI) and the risk of mortality among individuals with hypertension and to explore gender-specific effects.

**Methods:**

Data from the National Health and Nutrition Examination Survey (NHANES) from 1999 to 2018 were analyzed. Three models were constructed to assess the relationship between HGI and mortality risks, controlling for various covariates. Nonlinear relationships were explored using restricted cubic splines (RCS) and threshold effect analysis.

**Results:**

The findings reveal a U-shaped relationship between HGI and the cardiovascular disease (CVD) and all-cause mortality after adjusting for multiple covariates. Gender- specific analysis indicated a U-shaped relationship in men, with threshold points of -0.271, and 0.115, respectively. Before the threshold point, HGI was negatively associated with CVD mortality (HR: 0.64, 95%CI: 0.44, 0.93, P=0.02) and all-cause mortality (HR: 0.84, 95%CI: 0.71, 0.99), and after the threshold point, HGI was positively associated with CVD mortality (HR: 1.48, 95%CI: 1.23, 1.79, P<0.01) and all-cause mortality (HR: 1.41, 95%CI: 1.24, 1.60). In contrast, HGI had a J-shaped relationship with CVD mortality and a L-shaped relationship with all-cause mortality in females. Before the threshold points, the risk of all-cause mortality decreased (HR: 0.66, 95%CI:0.56, 0.77, P=0.04) and after the threshold points, the risk of CVD mortality increased (HR: 1.39, 95%CI:1.12, 1.72, P<0.01) progressively with increasing HGI.

**Conclusion:**

The research highlights the significance of maintaining proper HGI levels in individuals with hypertension and validates HGI as a notable indicator of cardiovascular and all-cause mortality risks. It also highlights the significant role of gender in the relationship between HGI and these risks.

## 1 Introduction

Hypertension is a prevalent chronic condition that has a significant impact on worldwide health and longevity. It is a major risk factor for cardiovascular diseases (CVD) and is a leading cause of death and disability worldwide (1, 2). Prompt identification and management of hypertension are crucial for improving health and reducing medical burdens, despite facing many challenges (3). Many patients are unaware of their hypertension until serious complications arise, leading to delayed or missed diagnoses (4, 5). Individual differences can cause patients to react differently to the same blood pressure medication (6), requiring tailored treatment approaches.

The Hemoglobin Glycation Index (HGI) measures how well an individual’s body processes glucose by comparing their actual glycated hemoglobin levels to what is expected (7). The HGI is calculated as the difference between an individual’s observed HbA1c and the HbA1c predicted by a linear regression equation based on the individual’s fasting plasma glucose (FPG) level (8). This regression equation is derived from a reference population and the HGI is expressed in units corresponding to a Gaussian distribution with a mean of 0 and a standard deviation of 1. This statistical method identifies individuals with HbA1c levels that deviate from expected values based on fasting glucose, accounting for biological variability in hemoglobin glycation. This variability, affected by factors like red blood cell lifespan and glycation rate, can cause discrepancies in diabetes management when using HbA1c alone. The connection between HGI and hypertension may be due to factors such as insulin resistance, a key mechanism in hypertension (9); second, its correlation with inflammation levels (10), a significant promoter of hypertension (11); and third, its association with oxidative stress (7), another critical factor in the development of hypertension. The HGI, reflecting cumulative glycemic exposure, could enhance understanding of long-term metabolic health in hypertensive individuals and complement traditional cardiovascular risk factors. Some studies have found a positive link between HGI and hypertension, but results vary (12). In a cross-sectional study, high HGI independently associated with risk of hypertension (13).In another cross-sectional study, HGI was also found to be predictive of the development of heart failure in a hypertensive population (14). Research on how HGI affects CVD and all-cause mortality in hypertensive patients is limited. Addressing this gap could enhance clinical practice by making HGI a valuable tool for early identification and optimization of interventions in high-risk hypertensive patients. This study aims to examine the relationship between HGI and the risk of cardiovascular and all-cause mortality in individuals with hypertension. By analyzing data from National Health and Nutrition Examination Survey (NHANES), we investigated the association between HGI and CVD and all-cause mortality in hypertension to provide valuable information for the prevention and management of hypertension.

## 2 Methods

### 2.1 Data source

This study used data from NHANES, a survey by NCHS (National Center for Health Statistics) in the US that evaluates the health and nutrition of Americans. The study used NHANES data from 1999 to 2018, consisting of ten two-year survey cycles. We followed NHANES guidelines to maintain data consistency and accuracy.

### 2.2 Sample selection

This study used the hypertension guidelines published by the American Heart Association in 2017 (15), which defined respondents with systolic blood pressure ≥140 mmHg or diastolic blood pressure ≥90 mmHg, or who were taking antihypertensive medication, as hypertensive. The study conducted blood pressure assessments using qualified personnel or physicians within mobile testing centers. Participants rested for 5 minutes before measurements were taken using a mercury sphygmomanometer. Three consecutive readings were obtained using an appropriately sized cuff on the right arm. An additional measurement was taken if a reading was disrupted or not fully captured. For our analysis, we calculated the mean of these readings for each participant. Participants who answered positively to any of the following questions were identified as having hypertension: “Have you ever been told by a doctor or other health professional that you have hypertension, also called high blood pressure?”; “are you now taking prescribed medicine for high blood pressure?”; or if they had a high biological measurement value (systolic blood pressure ≥ 140 mm Hg and/or diastolic blood pressure ≥ 90 mm Hg).

Based on this criterion, 22877 eligible hypertensive patients were screened from the NHANES 1999–2018 as the sample for analysis in this study. Since not all hypertensive patients had complete data for all variables in the NHANES data, only those hypertensive patients with complete data for all variables were selected for this study. After screening, 7607 people with hypertension were finally included (**Supplementary Figure 1**).

### 2.3 Parameter calculation: linear regression analysis of the relationship between FBG and HbA1c

**Figure 1:**
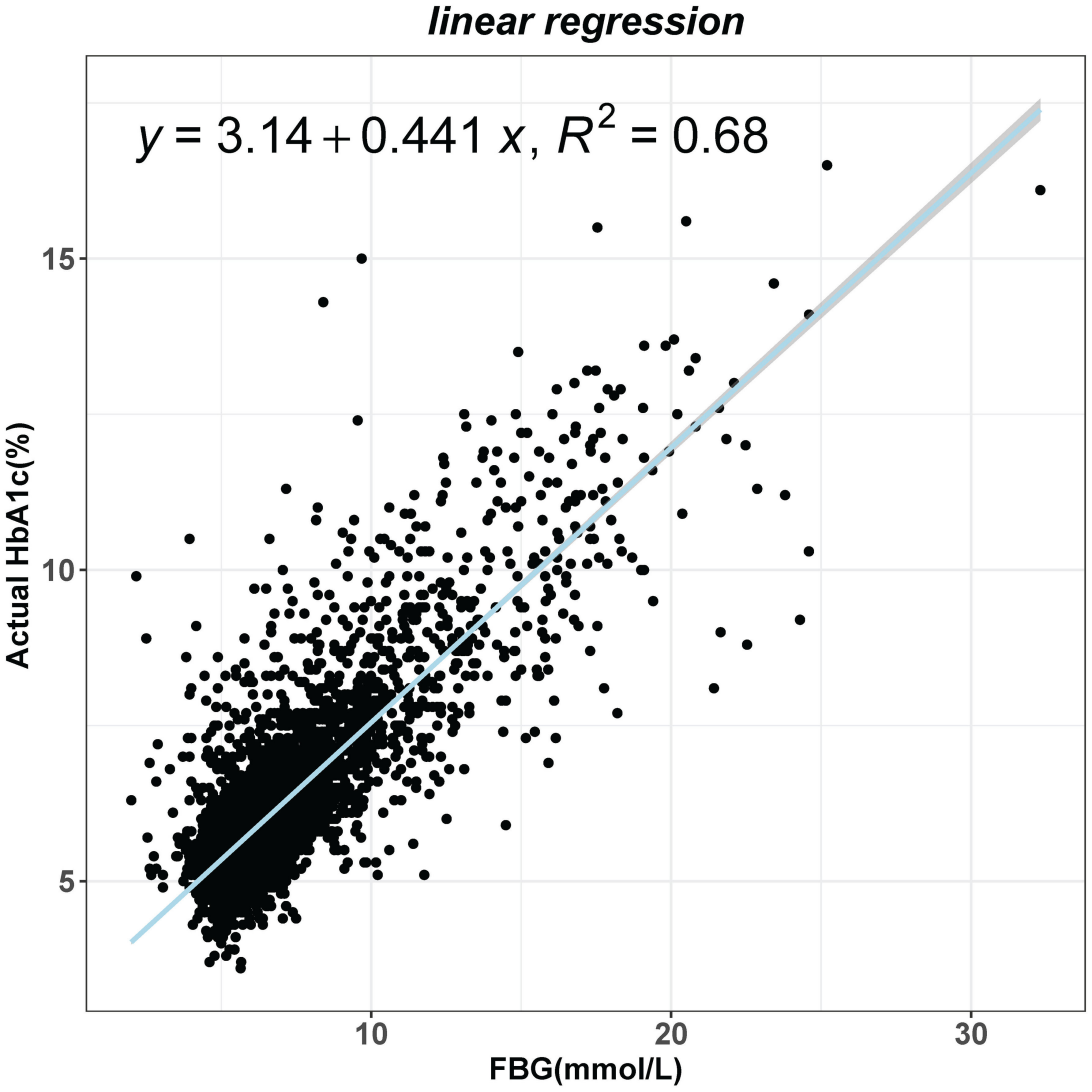
Linear regression analysis of the effect of fasting blood glucose (FBG) on glycated hemoglobin (HbA1c) levels.

The study calculated the HGI by subtracting the predicted HbA1c value from the actual HbA1c value. The relationship between FBG and actual HbA1c was analyzed using a linear regression model. The overall significance of the linear regression model was checked by F-test and it was found to be significant (F=16020, p<0.001). After extracting the residual values for each sample again, we found that the residual values were normally distributed by plotting the histogram (**Supplementary Figure 2A**) and normal QQ plot (**Supplementary Figure 2B**) of the residuals. Finally, several outliers were found in plotting the residual leverage plot (**Supplementary Figure 2C**). Due to our large sample size, the removal of the outliers did not affect the regression equation. Finally, a linear regression equation was obtained: predicting HbA1c = 0.441*FPG+3.14 (**Figure 1**).

### 2.4 Outcome

The study investigates cardiovascular and all-cause mortality among hypertensive individuals using NHANES follow-up data up to December 31, 2018, categorizing deaths via the NCHS system. All-cause mortality was determined using records from the National Death Index (NDI) up to December 31, 2019. These records were linked to the 1999–2018 NHANES data using an algorithm to confirm occurrences of death. Cardiovascular (I00-I09, I11, I13, I20-I51) mortality were defined according to the International Classification of Diseases, Tenth Clinical Revision (ICD-10) system codes and the NCHS classified heart diseases (054–064).

### 2.5 Assessment of covariates

We included a variety of covariates that could affect the results, including age, sex, race, education level, family poverty income ratio, smoking status, drinking status, body mass index (BMI), total cholesterol (TC), triglycerides (TG), high-density lipoprotein (HDL) cholesterol, low-density lipoprotein (LDL) cholesterol, included aspartate aminotransferase (AST), alanine aminotransferase (ALT), Serum creatinine (Scr), blood urea nitrogen (BUN). Data for these covariates were obtained from the NHANES questionnaire and laboratory test results(https://www.cdc.gov/nchs/nhanes/index.htm).

## 3 Statistical analysis

We used R software (version 4.3.0) and Empower (version 4.1) for data analysis. Continuous variables were described using means and standard deviations for normal distribution and medians and interquartile ranges for non-normal distribution. Categorical variables were reported with absolute frequencies and percentages. The t-test compared normally distributed variables, while the U and Kruskal-Wallis tests were used for non-normally distributed data. We categorized HGI into quintiles to assess its predictive value for all-cause and cardiovascular mortality, with Q0 and Q4 representing the lowest and highest levels. Cox proportional hazards regression analysis was conducted to assess the association between HGI and the endpoints. The Schoenfeld residual method is a type of residual used to test whether the residual terms of a Cox model are time-dependent. If the p-value of the test is >0.05 suggesting that the residual is stochastically related to time, it means that the proportional risk assumption is met. To mitigate confounding effects, multiple models were constructed. Model 1 was unadjusted; Model 2 adjusted for gender, age, and race; Model 3 expanded on Model 2 by adjusting for educational attainment, income ratio, BMI, lifestyle habits, lipid profiles, liver enzymes (ALT, AST), kidney function markers (BUN, serum creatinine), employing restricted cubic splines (RCS) for non-linear correlations and piecewise linear regression to identify inflection points, considering significant(P<0.05).

## 4 Results

### 4.1 Baseline characteristics of study participants

**Supplementary Table 1** shows the baseline characteristics of 7607 hypertensive patients, divided into quintiles based on the HGI. The average age of participants was 59.73 years, with males making up 51.77% of the group. Participants in the high HGI group were more likely to be female and non-Hispanic white. During a median follow-up period of 101 months, there were 531 CVD deaths and 1884 all-cause deaths. Participants with higher HGI were more likely to have obesity, high blood sugar, dyslipidemia, liver damage, and renal insufficiency compared to those with lower HGI. Additionally, significant differences in biochemical markers were found between the groups, with those in the highest quintile having higher levels of various markers compared to those in the lowest quintile(*P*<0.001).

### 4.2 Stratified effects of HGI quintiles on CVD and all-cause mortality risk

This study constructed three models to examine the relationship between the HGI and the risks of CVD and all-cause mortality. In the proportional risk assumption, the linear relationship between residuals and time was found to be insignificant (P>0.05) by the Schoenfeld residuals test, and the model met the proportional risk assumption (**Supplementary Figure 3**). After adjusting for multiple covariates, compared to the lowest quintile group (Q0), the highest quintile group (Q4) showed a significantly increased risk of cardiovascular disease mortality (HR: 1.34; 95% CI, 1.01–1.76) (**Table 1**). In contrast, all-cause mortality risk was significantly reduced in the Q2 (HR: 0.79; 95% CI, 0.69–0.92) and Q3 groups (HR: 0.86; 95% CI, 0.74–0.99) (**Table 1**).

**Table 1:** HRs (95% CIs) for mortality according to the HGI 5 quartiles.

	Model 1	Model 2	Model 3
CVD morality			
Q0	1.0	1.0	1.0
Q1	1.01 (0.77, 1.34) 0.92	1.02 (0.77, 1.35) 0.89	1.02 (0.77, 1.35) 0.91
Q2	1.04 (0.79, 1.38) 0.76	0.96 (0.72, 1.27) 0.76	0.95 (0.72, 1.26) 0.71
Q3	1.07 (0.81, 1.42) 0.63	0.96 (0.72, 1.27) 0.78	0.93 (0.70, 1.23) 0.61
Q4	1.53 (1.18, 2.00) <0.01	1.53 (1.16, 2.01) <0.01	1.34 (1.01, 1.76) 0.04
P for trend	<0.01	0.01	0.12
All-cause morality			
Q0	1.0	1.0	1.0
Q1	0.88 (0.77, 1.02) 0.08	0.88 (0.76, 1.01) 0.07	0.88 (0.76, 1.01) 0.08
Q2	0.87 (0.75, 1.00) 0.05	0.79 (0.69, 0.91) <0.01	0.79 (0.69, 0.92) <0.01
Q3	0.97 (0.84, 1.12) 0.65	0.86 (0.74, 0.99) 0.04	0.86 (0.74, 0.99) 0.03
Q4	1.09 (0.94, 1.25) 0.25	1.05 (0.90, 1.21) 0.55	0.99 (0.85, 1.14) 0.87
P for trend	0.13	0.83	0.64

Model 1 adjusted for: None.

Model 2 adjusted for: Gender; Age; Race.

Model 3 adjusted for: Gender; Age; Race; Education; PIR; BMI; Drinking; Smoking; TG; TC; HDL; LDL; ALT; AST; BUN; Scr.

HGI, hemoglobin glycation index; HR, Hazard ratio; CI, Confidence interval.

### 4.3 Threshold effect analysis of HGI levels and their impact on mortality risk

**Figure 2:**
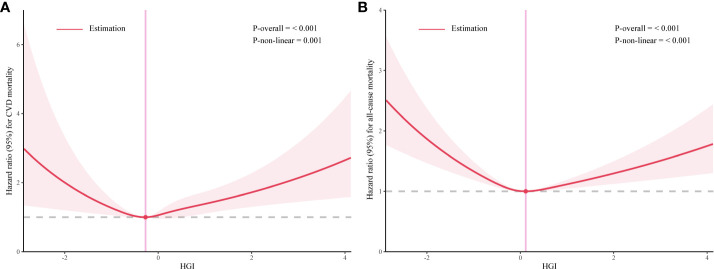
The association between high glycemic index (HGI) and mortality rates related to cardiovascular events **(A)** and all-cause **(B)**.

Our analysis initially indicated a potential non-linear relationship between the HGI and both CVD mortality and all-cause mortality rates. Utilizing RCS and threshold effect analysis, we identified an inflection point in the impact of HGI on both all-cause (**Figure 2A**) and CVD mortality (**Figure 2B**). For all-cause mortality, on the left side of the inflection point=0.115, the risk of death decreased with increasing HGI, with a multivariable-adjusted hazard ratio (HR) of 0.75 (95% CI=0.67–0.84, P<0.01, **Table 2**). Conversely, when HGI was greater than or equal to 0.115, a higher HGI was associated with an increased risk of death, with HR being 1.24 (95% CI=1.14–1.35, P<0.01, **Table 2**). For CVD mortality, the inflection point was -0.271. When HGI was less than -0.271, an increase in HGI was associated with a reduced risk of death, with HR being 0.65 (95% CI=0.57–0.75, P<0.01, **Table 2**); when HGI was greater than or equal to -0.271, an increase in HGI correlates with an increased risk of death, with HR being 1.19 (95% CI=1.10–1.28, P<0.01, **Table 2**). This suggests that the relationship between HGI and mortality risk was not simply linear but exhibits a U-shaped relationship. Relevant data are presented in **Table 3**.

**Table 2:** Threshold effect analysis of HGI on all-cause and CVD mortality.

	Adjusted HR (95% CI)	P-value
All-cause mortality		
Inflection point	0.115	
HGI < 0.115	0.75 (0.67, 0.84)	<0.01
HGI ≥ 0.115	1.24 (1.14, 1.35)	<0.01
P for Log-likelihood ratio	< 0.01	
CVD mortality		
Inflection point	-0.271	
HGI < -0.271	0.65 (0.57, 0.75)	<0.01
HGI ≥ -0.271	1.19 (1.10, 1.28)	<0.01
P for Log-likelihood ratio	< 0.01	

Cox proportional hazards models were used to estimate HR and 95% CI. Adjusted for gender, age, race, education, PIR, BMI, drinking, smoking, TG, TC, HDL, LDL, ALT, AST, BUN, Scr.

**Table 3:** Subgroup analysis based on gender.

Exposure	Male	female	P for interaction
CVD mortality			0.50
Inflection point= -0.271			
HGI < -0.271	0.64 (0.44, 0.93) 0.02	0.68 (0.42, 1.10) 0.12	
HGI ≥ -0.271	1.48 (1.23, 1.79) < 0.01	1.39 (1.12, 1.72) < 0.01	
P for Log-likelihood ratio	< 0.01	0.03	
All-cause mortality			< 0.01
Inflection point=0.115	0.03	0.87	
HGI < 0.115	0.84 (0.71,0.99) 0.04	0.66 (0.56,0.77) 0.04	
HGI ≥ 0.115	1.41 (1.24, 1.60) <0.01	1.11 (0.97, 1.26) 0.13	
P for Log-likelihood ratio	<0.01	<0.01	

Cox proportional hazards models were used to estimate HR and 95% CI. Adjusted for age, race, education, PIR, BMI, drinking, smoking, TG, TC, HDL, LDL, ALT, AST, BUN, Scr.

### 4.4 Gender differences in the relationship between HGI levels and mortality

We re-evaluated the relationship between HGI levels and the risks of cardiovascular and all-cause mortality in patients with hypertension, specifically focusing on gender-specific data. This analysis revealed distinct relationship patterns under the lens of gender differences (**Table 3**). In men, the analysis demonstrated a U-shaped relationship between HGI and mortality risk. Specifically, the HR (95%CI) for CVD mortality was 0.64 (0.44, 0.93) and all-cause mortality was 0.84 (0.71,0.99), and for the right side was 1.48 (1.23, 1.79) and 1.41 (1.24, 1.60), respectively. This indicates that both extremely low and high levels of HGI contribute to elevated mortality risks in men.

For women, a J-shaped (or threshold effect) relationship between HGI and cardiovascular mortality risk was observed. Notably, an increase in cardiovascular mortality risk significantly correlated with HGI levels above -0.271 (HR=1.39, 95% CI= 1.12–1.72, P<0.01, **Table 3**). In the context of all-cause mortality, women exhibited an L-shaped (or saturation effect) relationship. On the left of the inflection point, there was a reduction in all-cause mortality risk (HR=0.66, 95% CI=0.56–0.77, P=0.04, **Table 3**), whereas the trend of increased mortality risk was not significantly evident when HGI exceeded 0.115 (HR=1.11, 95% CI=0.97–1.26, P=0.13, **Table 3**).

### 4.5 Sensitivity analysis

We confirmed the U-shaped relationship of the HGI with CVD and all-cause mortality through sensitivity analyses that took into account participants who died within two years prior to follow-up and participants with diabetes. Findings remained unchanged after adjusting for potential confounders, underscoring the credibility of our primary outcomes (**Supplementary Table 2**).

## 5 Disucssion

This study examined the nonlinear relationship between HGI and cardiovascular and all-cause mortality rates in patients with hypertension using analysis of NHANES data. The study found a U-shaped correlation between HGI and mortality in hypertensive patients, showing that optimal HGI levels minimize cardiovascular risk, highlighting HGI’s importance as a mortality predictor.

Several studies have yielded results similar to ours. Zeng and colleagues found a U-shaped relationship between HbA1c levels and mortality rates in patients with hypertension. Below a certain threshold, increasing HGI levels reduce mortality risk, but above that threshold, they increase mortality risk (16). Jian-di Wu’s team discovered that high HGI is linked to higher risks of CVD and mortality, especially when HbA1c levels are considered (17). Kim MK and others concluded that HGI is independently associated with CVD events in patients with type 2 diabetes, with patients having high HGI at baseline possessing a higher inherent risk of CVD (18). Moreover, Cheng and colleagues analyzed the association between HGI and the long-term prognosis of patients with coronary artery disease (CAD) after percutaneous coronary intervention (PCI). They observed that, compared to patients with moderate HGI, those with low and high HGI had a significantly increased risk of all-cause mortality (19). Possible explanations for these findings include: Firstly, Higher HGI can signal metabolic instability linked to insulin resistance, blood sugar fluctuations, energy metabolism disorders, and oxidative stress, a major risk factor for cardiovascular diseases (20). Oxidative stress, characterized by an imbalance between oxidants and antioxidants within the body, results in cellular damage and dysfunction, thereby serving as a significant risk factor for cardiovascular disease (21). Lastly, these factors, such as insulin resistance and oxidative stress, may collectively lead to endothelial dysfunction and increase the risk of atherosclerosis (22, 23). Chronic inflammation can also promote vascular inflammation and plaque formation, further increasing the risk of cardiovascular events (24).

Alternatively, Jing-Lu Jin proposed that for patients with newly diagnosed stable CAD and type 2 diabetes mellitus (T2DM), the TyG index might have a better prognostic value than HGI (25). Other studies suggest that, akin to HbA1c, elevated HGI links to increased cardiovascular risk in T2DM patients without prior CVD. Given HbA1c’s established risk role and the challenges in assessing and interpreting HGI, its extra clinical utility may be minimal (26). van Steen SC further pointed out that given the inconsistent results and uncertain correlations beyond HbA1c, the clinical use of HGI in type 2 diabetes is currently not recommended (27). Inconsistencies may arise from population diversity (variations in geography, ethnicity, age affecting outcomes due to environmental and genetic influences) and research methods (data collection, analysis techniques, and adjustments for confounders significantly impact result consistency). Statistical considerations: The size of the sample, choice of statistical methods, and their power could lead to significant differences in outcomes between studies; Research quality and bias: The quality of design and process bias directly affect the credibility and consistency of study results.

In this study, we constructed three models (28) to thoroughly explore the relationship between the HGI and both cardiovascular mortality and all-cause mortality rates among patients with hypertension. The findings reveal that after adjusting for multiple covariates, the highest HGI quintile group (Q4) exhibited a significantly increased rate of cardiovascular disease mortality compared to the lowest HGI quintile group (Q0) (HR: 1.34; 95% CI, 1.01–1.76). All-cause mortality rates were significantly reduced in the Q2 and Q3 groups. These findings hold significant clinical implications for the management of hypertension. Analyzing gender differences, we discovered a U-shaped link between HGI and mortality risks in men. A J-shaped (or threshold effect) relationship between HGI and cardiovascular mortality risk and an L-shaped (or saturation effect) relationship with all-cause mortality were found. Moderate HGI levels in women were linked to decreased cardiovascular and mortality risks, with estrogen potentially safeguarding women’s heart health (29). Moreover, there are differences in energy and lipid metabolism between men and women (24). For instance, men have a higher tendency to store visceral fat, whereas women typically have more subcutaneous fat buildup (30). Visceral fat is linked to insulin resistance, inflammation, and a higher likelihood of developing cardiovascular diseases (31, 32). Hormonal variations may clarify the diminished mortality risk from elevated HGI in women. Limited gender difference research necessitates further study to grasp its impact on cardiovascular health.

### 5.1 Strengths and limitations

The study’s strength lies in its extensive sample size and extended follow-up period, improving the statistical reliability and generalizability of our findings. Moreover, thorough adjustment for confounding variables enhances the precision of our analysis results, providing a more accurate reflection of the association between HGI and cardiovascular and all-cause mortality risk. Moreover, these results have practical implications for primary care practice. Primary care physicians may find it beneficial to use HGI as a prognostic tool when treating hypertensive patients. This could lead to better outcomes by categorizing risk according to HGI levels. However, there are some limitations of our study. Firstly, the parameters used to calculate the HGI, FPG and glycated hemoglobin, had missing partial data that may have an impact on mortality outcomes in diabetic populations. Second, the calculation of the HGI index was based on single measurements of FPG and HbA1c, which do not reflect dynamic changes during long-term follow-up. Third, our study population was from the US population, which may limit the generalizability of our findings to other ethnic groups or other populations.

## 6 Conclusion

In conclusion, this study demonstrated a U-shaped relationship between HGI and cardiovascular disease mortality and all-cause mortality in a hypertensive population. It is important for individuals with hypertension to regularly monitor their HGI and make necessary adjustments to their treatment plans and recommended to maintain stable levels when HGI is moderate in order to minimize mortality risk.

## Data availability statement

Publicly available datasets were analyzed in this study. This data can be found here: https://www.cdc.gov/nchs/nhanes/index.htm.

## Ethics statement

The studies were conducted in accordance with the local legislation and institutional requirements. Written informed consent for participation was not required from the participants or the participants' legal guardians/next of kin in accordance with the national legislation and institutional requirements.

## Author contributions

QS: Writing – original draft, Conceptualization, Data curation, Investigation, Methodology. JY: Writing – original draft, Conceptualization, Methodology, Supervision. BL: Writing – original draft, Data curation, Methodology, Software, Validation. HC: Writing – original draft, Conceptualization, Data curation, Methodology, Software. LY: Writing – review & editing, Supervision, Validation.

## References

[1] Hunter PG Chapman FA Dhaun N. Hypertension: Current trends and future perspectives. Br J Clin Pharmacol. (2021) 87:3721–36. doi: 10.1111/bcp.14825 33733505

[2] Mills KT Stefanescu A He J. The global epidemiology of hypertension. Nat Rev Nephrol. (2020) 16:223–37. doi: 10.1038/s41581-019-0244-2 PMC7998524 32024986

[3] Carey RM Wright JT Jr Taler SJ Whelton PK. Guideline-driven management of hypertension: an evidence-based update. Circ Res. (2021) 128:827–46. doi: 10.1161/CIRCRESAHA.121.318083 PMC8034801 33793326

[4] Davari M Sorato M Nikfar S Sorato M. Cost-effectiveness of comprehensive screening of general population for hypertension: can it save money and life? Systematic Rev Pharmacoeconomic Stud. (2020) 19:126–6. doi: 10.21203/rs.2.24367/v1

[5] Gauer R. Severe asymptomatic hypertension: evaluation and treatment. Am Family Physician. (2017) 95:492–500 28409616

[6] Kutumova E Kiselev I Sharipov RN Lifshits G Kolpakov F. Mathematical modeling of antihypertensive therapy. Front Physiol. (2022) 13:1070115. doi: 10.3389/fphys.2022.1070115 36589434 PMC9795234

[7] Lyu L Yu J Liu Y He S Zhao Y Qi M. High hemoglobin glycation index is associated with telomere attrition independent of HbA1c, mediated by TNFα. J Clin Endocrinol Metab. (2022) 107:462–73. doi: 10.1210/clinem/dgab703 34562085

[8] Hempe JM Yang S Liu S Hsia DS. Standardizing the haemoglobin glycation index. Endocrinol Diabetes Metab. (2021) 4:e00299. doi: 10.1002/edm2.299 34558807 PMC8502217

[9] Hill MA Yang Y Zhang L Sun Z Jia G Parrish AR. Insulin resistance, cardiovascular stiffening, and cardiovascular disease. Metabolism-Clinical Experimental. (2021) 119:154766. doi: 10.1016/j.metabol.2021.154766 33766485

[10] Lyu L Yu J Liu Y He S Zhao Y Qi M. Dietary patterns, oxidative Stress, inflammation and biological variation in hemoglobin A1c: Association and Mediation analysis in a rural community in north China. Diabetes Res Clin Practice. (2022) 194:110154. doi: 10.1016/j.diabres.2022.110154 36379413

[11] Lazaridis A Gavriilaki E Dolgyras P Koletsos N Triantafyllou A Anyfanti P. HIGH MOBILITY GROUP BOX-1 IS INCREASED IN NEWLY DIAGNOSED ESSENTIAL HYPERTENSIVES AND CORRELATES WITH HYPERTENSION-MEDIATED TARGET ORGAN DAMAGE. J Hypertension. (2022) 40:e44. doi: 10.1097/01.hjh.0000835632.02104.9d

[12] Mi J Song J Zhao Y Wu XS. Association of hemoglobin glycation index and its interaction with obesity/family history of hypertension on hypertension risk: a community-based cross-sectional survey. BMC Cardiovasc Disord. (2020) 20:0–0. doi: 10.1186/s12872-020-01762-0 PMC7640660 33148181

[13] Wang R Chen C Xu G ZN J. Association of triglyceride glucose-body mass index and hemoglobin glycation index with heart failure prevalence in hypertensive populations: a study across different glucose metabolism status. Lipids Health Dis. (2024) 23:53. doi: 10.1186/s12944-024-02045-9 38388437 PMC10882741

[14] Seen TK Sayed M Bilal M Reyes JV Bhandari P Lourdusamy V. Clinical indicators for progression of nonalcoholic steatohepatitis to cirrhosis. World J Gastroenterol. (2021) 27:3238–48. doi: 10.3748/wjg.v27.i23.3238 PMC8218360 34163108

[15] Whelton PK Carey RM Wilbert S Casey DE Jr Collins KJ Himmelfarb CD. 2017 ACC/AHA/AAPA/ABC/ACPM/AGS/APhA/ASH/ASPC/NMA/PCNA guideline for the prevention, detection, evaluation, and management of high blood pressure in adults: A report of the American college of cardiology/American heart association task force on clinical practice guidelines. Circulation. (2018) 138:e484–594. doi: 10.1161/CIR.0000000000000596 30354654

[16] Zeng RX Zhang YZ Xu JP Kong Y Tan J Guo L. Relationship of glycated hemoglobin A1c with all-cause and cardiovascular mortality among patients with hypertension. J Clin Med. (2023) 12:2615. doi: 10.3390/jcm12072615 37048698 PMC10095266

[17] Wu J Liang D Xie Y Chen M-y Chen H-h Sun D. Association between hemoglobin glycation index and risk of cardiovascular disease and all-cause mortality in type 2 diabetic patients: A meta-analysis. Front Cardiovasc Med. (2021) 8:690689. doi: 10.3389/fcvm.2021.690689 34124211 PMC8193090

[18] Kim MK Jeong JS Yun JS Kwon HS Baek KH Song KH. Hemoglobin glycation index predicts cardiovascular disease in people with type 2 diabetes mellitus: A 10-year longitudinal cohort study. J Diabetes Complications. (2018) 32:906–10. doi: 10.1016/j.jdiacomp.2018.07.004 30121206

[19] Cheng MD Tang J Liu Z Guo Q-Q Zhang J-C Zhang Z-L. Association of hemoglobin glycation index with prognosis of coronary artery disease after percutaneous coronary intervention: A retrospective cohort study. Diabetes Vasc Dis Res. (2023) 20:0–0. doi: 10.1177/14791641211056874 PMC10416663 37561132

[20] Marini MA Fiorentino TV Succurro E Pedace E Andreozzi F Sciacqua A. Association between hemoglobin glycation index with insulin resistance and carotid atherosclerosis in non-diabetic individuals. PloS One. (2017) 12:e0175547. doi: 10.1371/journal.pone.0175547 28426788 PMC5398507

[21] Srivastava R. Lifestyle-induced metabolic derangement and epigenetic changes promote diabetes and oxidative stress leading to NASH and atherosclerosis severity. J Diabetes Metab Disord. (2018) 17:381–91. doi: 10.1007/s40200-018-0378-y PMC6405391 30918873

[22] Maruhashi T Higashi Y. Pathophysiological association between diabetes mellitus and endothelial dysfunction. Antioxidants. (2021) 10:1306. doi: 10.3390/antiox10081306 34439553 PMC8389282

[23] Maamoun H Abdelsalam SS Zeidan A Korashy HM Agouni A. Endoplasmic reticulum stress: A critical molecular driver of endothelial dysfunction and cardiovascular disturbances associated with diabetes. Int J Mol Sci. (2019) 20:1658. doi: 10.3390/ijms20071658 30987118 PMC6480154

[24] de Ritter R Sep SJS van Greevenbroek MMJ Kusters YHAM Vos RC Bots ML. Sex differences in body composition in people with prediabetes and type 2 diabetes as compared with people with normal glucose metabolism: the Maastricht Study. Diabetologia. (2023) 66:861–72. doi: 10.1007/s00125-023-05880-0 PMC10036428 36805778

[25] Jin JL Sun D Cao YX Guo YL Wu NQ Zhu CG. Triglyceride glucose and haemoglobin glycation index for predicting outcomes in diabetes patients with new-onset, stable coronary artery disease: a nested case-control study. Ann Med. (2018) 50:576–86. doi: 10.1080/07853890.2018.1523549 30207490

[26] Østergaard HB Poulsen TM Berkelmans GFN van der Graaf Y Visseren FLJ Westerink J. Limited benefit of haemoglobin glycation index as risk factor for cardiovascular disease in type 2 diabetes patients. Diabetes Metab. (2019) 45:254–60. doi: 10.1016/j.diabet.2018.04.006 29784563

[27] van Steen SC Woodward M Chalmers J Li Q Marre M Cooper ME. Haemoglobin glycation index and risk for diabetes-related complications in the Action in Diabetes and Vascular Disease: Preterax and Diamicron Modified Release Controlled Evaluation (ADVANCE) trial. Diabetologia. (2018) 61:780–9. doi: 10.1007/s00125-017-4539-1 PMC6448976 29308539

[28] Zhang Q Xiao S Jiao X Shen Y. The triglyceride-glucose index is a predictor for cardiovascular and all-cause mortality in CVD patients with diabetes or pre-diabetes: evidence from NHANES 2001–2018. Cardiovasc Diabetol. (2023) 22:279. doi: 10.1186/s12933-023-01648-5 37848879 PMC10583314

[29] Mathur P Ostadal B Romeo F Mehta JL. Gender-related differences in atherosclerosis. Cardiovasc Drugs Ther. (2015) 29:319–27. doi: 10.1007/s10557-015-6596-3 26006701

[30] Fox CS Liu Y White CC Feitosa M Smith AV Heard-Costa N. Genome-wide association for abdominal subcutaneous and visceral adipose reveals a novel locus for visceral fat in women. PLoS Genet. (2012) 8:e1002695. doi: 10.1371/journal.pgen.1002695 22589738 PMC3349734

[31] Lim S Meigs JB. Links between ectopic fat and vascular disease in humans. Arterioscler Thromb Vasc Biol. (2014) 34:1820–6. doi: 10.1161/ATVBAHA.114.303035 PMC4140970 25035342

[32] Karlsson T Rask-Andersen M Pan G Höglund J Wadelius C Ek WE. Contribution of genetics to visceral adiposity and its relation to cardiovascular and metabolic disease. Nat Med. (2019) 25:1390–5. doi: 10.1038/s41591-019-0563-7 31501611

