# Supplementary material for: Association of the hemoglobin glycation index with cardiovascular and all-cause mortality in individuals with hypertension: findings from NHANES 1999–2018
Source: Front Endocrinol (Lausanne). 2024 Jun 10;15:1401317. doi: 10.3389/fendo.2024.1401317 (PMC11194314; doi:10.3389/fendo.2024.1401317)
Supplement: Supplementary file 1 [file DataSheet_1.pdf]

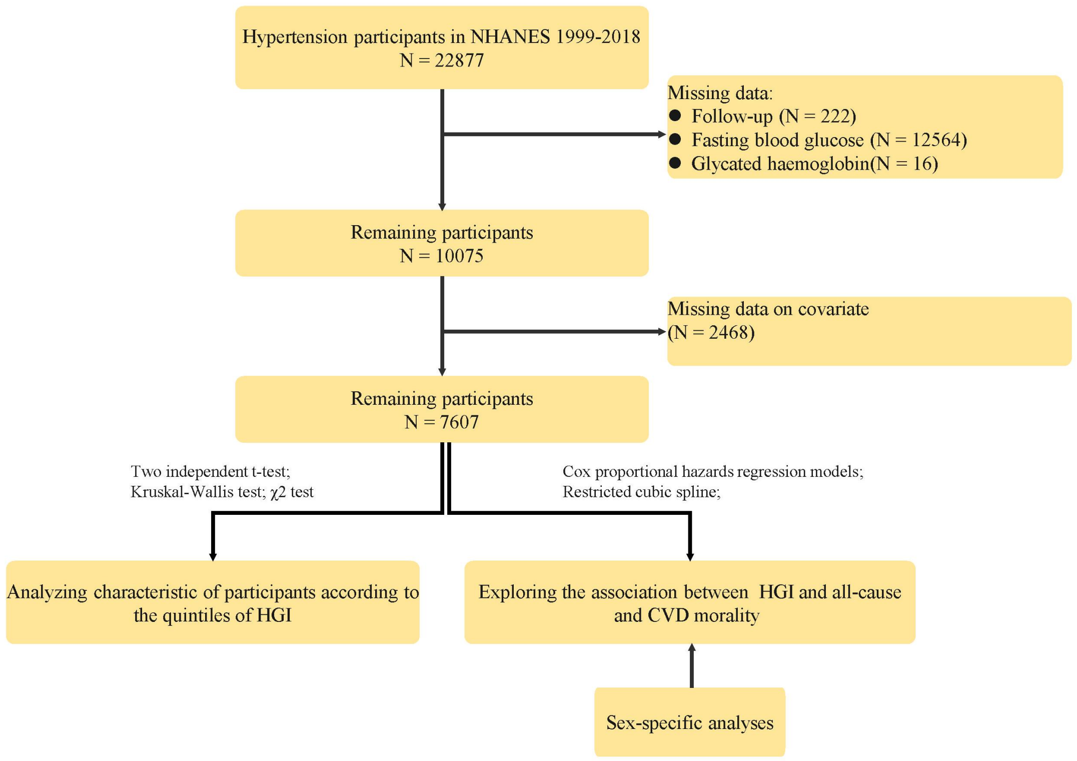

Supplementary Figure 1. Flow chart of the study on the association between HGI and mortality in patients with hypertension.

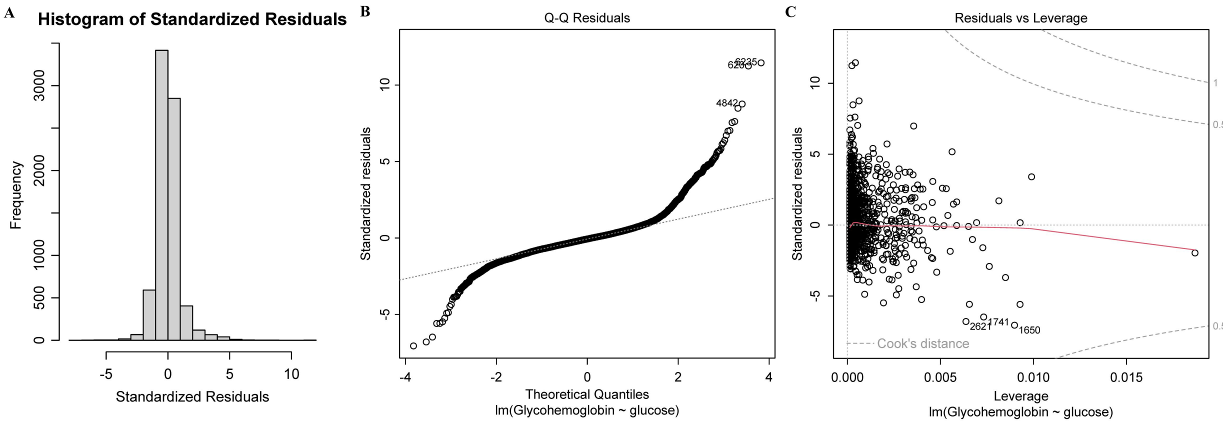

Supplementary Figure 2. Evaluation of linear regression models.

A.Histogram of standardised residuals. B. Normal QQ plot of the residuals. C. Leverage plot of the residuals.

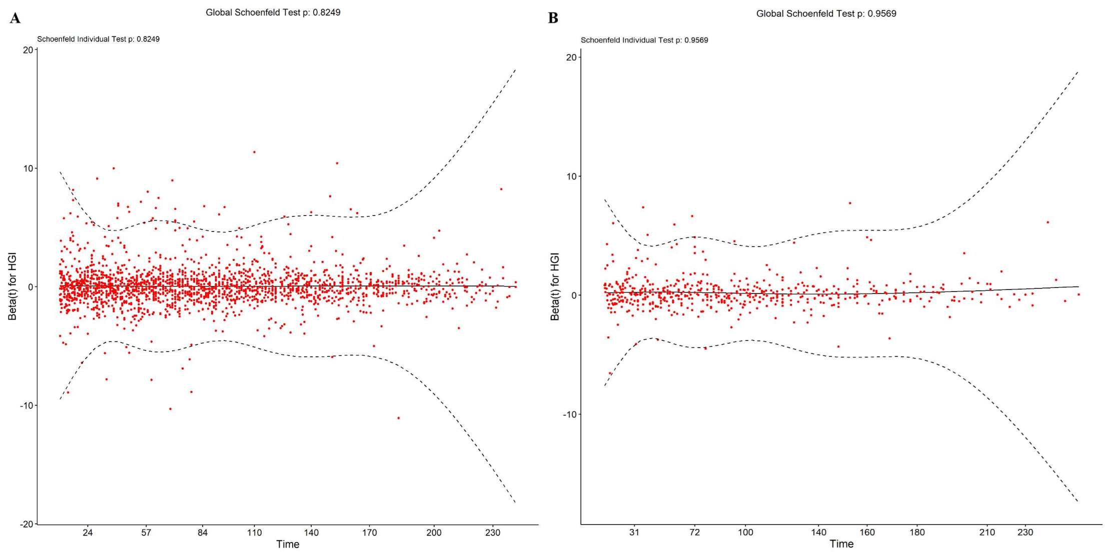

Supplementary Figure 3. Evaluation of the Cox proportional hazards model by Schoenfeld residual method

A. Cox proportional hazards model for HGI and CVD mortality assessed by Schoenfeld residual method. B. Cox proportional hazards model for HGI and all-cause mortality assessed by Schoenfeld residual method.

Supplementary Table 1. Baseline characteristics

HGI 5 Quartiles	Q0 (-4.65 - -0.41)	Q1 (-0.41- -0.15)	Q2 (-0.15-0.06)	Q3 (0.06-0.33)	Q4 (0.33- 7.59)	P-value
N	1522	1521	1504	1538	1522	
BMI	30.38 ± 7.50	30.27 ± 6.88	30.24 ± 7.15	30.38 ± 7.00	32.20 ± 7.70	<0.01
Age	57.69 ± 16.57	58.21 ± 16.16	59.61 ± 15.66	61.47 ± 14.92	61.67 ± 13.17	<0.01
Glucose	7.05 ± 2.75	6.09 ± 1.45	5.98 ± 1.47	5.93 ± 1.60	6.95 ± 2.86	<0.01
Glycohemoglobin	5.50 ± 1.04	5.55 ± 0.64	5.73 ± 0.65	5.94 ± 0.71	7.09 ± 1.65	<0.01
HDL	1.36 ± 0.46	1.38 ± 0.42	1.41 ± 0.43	1.42 ± 0.41	1.35 ± 0.39	<0.01

AST	27.68 ± 19.52	26.05 ± 16.30	25.99± 31.84	24.97 ± 11.35	25.03 ± 13.31	<0.01
ALT	27.62 ± 21.30	25.76 ± 19.28	24.61 ± 19.04	23.70 ± 14.56	24.33 ± 14.24	<0.01
BUN	5.06 ± 2.66	4.94 ± 2.59	5.03 ± 2.37	5.08 ± 2.44	5.44 ± 2.98	<0.01
Scr	88.51 ± 57.16	83.84 ± 64.23	82.06 ± 34.67	84.27 ± 49.65	90.50 ± 68.13	<0.01
TC	4.94 ± 1.04	5.15 ± 1.08	5.11 ± 1.07	5.14 ± 1.10	4.91 ± 1.13	<0.01
LDL	2.88 ± 0.90	3.06 ± 0.94	3.02 ± 0.93	3.04 ± 0.97	2.87 ± 1.00	<0.01
TG	1.52 ± 0.82	1.54 ± 0.77	1.48 ± 0.77	1.47 ± 0.77	1.49 ± 0.78	0.02
Gender						<0.01
Male	916 (60.18%)	792 (52.07%)	726 (48.27%)	655 (42.59%)	667 (43.82%)	
Female	606 (39.82%)	729 (47.93%)	778 (51.73%)	883 (57.41%)	855 (56.18%)	
Race						<0.01
Mexican American	176 (11.56%)	239 (15.71%)	222 (14.76%)	199 (12.94%)	230 (15.11%)	
Other Hispanic	97 (6.37%)	102 (6.71%)	114 (7.58%)	91 (5.92%)	119 (7.82%)	
Non-Hispanic White	892 (58.61%)	857 (56.34%)	796 (52.93%)	705 (45.84%)	460 (30.22%)	
Non-Hispanic Black	269 (17.67%)	214 (14.07%)	290 (19.28%)	429 (27.89%)	585 (38.44%)	
Other Race	88 (5.78%)	109 (7.17%)	82 (5.45%)	114 (7.41%)	128 (8.41%)	
Drinking						<0.01
Never	476 (31.27%)	511 (33.60%)	561 (37.30%)	678 (44.08%)	724 (47.57%)	
Every day or	229 (15.05%)	241 (15.84%)	220 (14.63%)	238 (15.47%)	234 (15.37%)	

nearly every day						
3 to 4 times a week	221 (14.52%)	203 (13.35%)	183 (12.17%)	176 (11.44%)	166 (10.91%)	
1 to 2 times a week	466 (30.62%)	442 (29.06%)	422 (28.06%)	325 (21.13%)	269 (17.67%)	
Less than once a week	130 (8.54%)	124 (8.15%)	118 (7.85%)	121 (7.87%)	129 (8.48%)	
Smoking						<0.01
Never	722 (47.44%)	726 (47.73%)	718 (47.74%)	806 (52.41%)	788 (51.77%)	
Now	265 (17.41%)	291 (19.13%)	287 (19.08%)	245 (15.93%)	283 (18.59%)	
Former	535 (35.15%)	504 (33.14%)	499 (33.18%)	487 (31.66%)	451 (29.63%)	
Education						<0.01
Less than high school	387 (25.43%)	409 (26.89%)	414 (27.53%)	439 (28.54%)	499 (32.79%)	
High or equivalent	364 (23.92%)	363 (23.87%)	370 (24.60%)	399 (25.94%)	403 (26.48%)	
College or above	771 (50.66%)	749 (49.24%)	720 (47.87%)	700 (45.51%)	620 (40.74%)	
PIR						<0.01
<=1	273 (17.94%)	263 (17.29%)	267 (17.75%)	276 (17.95%)	352 (23.13%)	
>1, <=3	629 (41.33%)	679 (44.64%)	651 (43.28%)	708 (46.03%)	708 (46.52%)	
>3	620 (40.74%)	579 (38.07%)	586 (38.96%)	554 (36.02%)	462 (30.35%)	

Data were presented as mean ± standard deviation or n (%). BMI: Body Mass Index; PIR: Poverty

Impact Ratio; LDL: Low-density lipoprotein; HDL: High-density lipoprotein; TC: Total Cholesterol; TG: Triglycerides; AST: Aspartate transaminase; ALT: Alanine aminotransferase; BUN: Blood urea nitrogen; Scr: Serum creatinine.

Supplementary Table 2. Sensitivity Analysis of HGI's Effect on All-Cause and CVD Mortality at Established Thresholds

	Sensitivity-1	Sensitivity-2	Sensitivity-3
CVD mortality			
Inflection point= -0.271			
HGI < -0.271	0.71 (0.53, 0.98) 0.04	0.70 (0.51, 0.97) 0.03	0.54 (0.29, 0.99) 0.04
HGI ≥ -0.271	1.37 (1.20, 1.58) <0.01	1.30 (1.11, 1.53) 0.01	1.76 (1.35, 2.29) <0.01
P for Log-likelihood ratio	0.01	0.01	0.01
All-cause mortality			
Inflection point=0.115			
HGI < 0.115	0.76 (0.67, 0.87) <0.01	0.77 (0.67, 0.88) <0.01	0.66 (0.52, 0.84) <0.01
HGI ≥ 0.115	1.24 (1.13, 1.36) <0.01	1.18 (1.06, 1.31) <0.01	1.57 (1.31, 1.88) <0.01

P for Log-likelihood ratio	<0.01	<0.01	<0.01

Sensitivity-1: After excluding participants who died within the first two years of follow-up, 7362 participants were included, adjusted for age, gender, race, education, PIR, BMI, drinking, smoking, TG, TC, HDL, LDL, ALT, AST, BUN, Scr;

Sensitivity-2: including 2234 participants with diabetes, adjusted for age, race, education, PIR, BMI, drinking, smoking, TG, TC, HDL, LDL, ALT, AST, BUN, Scr;

Sensitivity-3: including 3277 participants with age < 60 year, adjusted for age, race, education, PIR, BMI, drinking, smoking, TG, TC, HDL, LDL, ALT, AST, BUN, Scr;
